# Managing mental health: why we need to redress the balance between healthcare spending and social spending

**DOI:** 10.1186/s12889-020-08491-1

**Published:** 2020-03-26

**Authors:** Daniel S. Park, Jing Han, Mahmoud Torabi, Evelyn L. Forget

**Affiliations:** 1 Somerville College, University of Oxford, Woodstock Road, Oxford, OX26HD UK; 2 Rady Faculty of Health Sciences, University of Manitoba, 727 McDermot Avenue, Winnipeg, MB R3E 3P5 Canada

**Keywords:** Mental health, OECD, Social spending, Healthcare spending, Mental and behavioural disorders, Social determinants, Population health

## Abstract

**Background:**

Mental health outcomes vary widely among high-income countries, although mental health problems represent an increasing proportion of the burden of disease for all countries. This has led to increased demand for healthcare services, but mental health outcomes may also be particularly sensitive to the availability of social services. This paper examines the variation in the absolute and relative amounts that high-income countries spend on healthcare and social services to determine whether increased expenditure on social services relative to healthcare expenditure might be associated with better mental health outcomes.

**Methods:**

This paper estimates the association between patterns of government spending and population mental health, as measured by the death rate resulting from mental and behavioural disorders, across member countries of the Organisation for Economic Cooperation and Development (OECD). We use country-level repeated measures multivariable modelling for the period from 1995 to 2016 with region and time effects, adjusted for total spending and demographic and economic characteristics. Healthcare spending includes all curative services, long-term care, ancillary services, medical goods, preventative care and administration whilst social spending consists of all transfer payments made to individuals and families as part of the welfare state.

**Results:**

We find that a higher ratio of social to healthcare expenditure is associated with significantly better mental health outcomes for OECD populations, as measured by the death rate resulting from mental and behavioural disorders. We also find that there is no statistically significant association between healthcare spending and population mental health when we do not control for social spending.

**Conclusion:**

This study suggests that OECD countries can have a significant impact on population mental health by investing a greater proportion of total expenditure in social services.

## Background

All member countries of the Organisation for Economic Cooperation and Development (OECD) have experienced increases in the real cost of healthcare over the last 20 years. The average OECD country has seen healthcare costs increase by more than 2% of gross domestic product (GDP) since 1990, compared to a rise of 0.4% between 1980 and 1990 [1]. Globally, there has been a substantial decline in the overall death rate due to advances in medical treatment for all major physical health problems including cancer, cardiovascular diseases and strokes [2].

Despite this, there has been a rapid increase in the death rate due to mental and behavioural disorders, with the OECD average more than tripling between 1980 and 2015 (Table 1).

**Table 1:** Mortality across the OECD (deaths per 100,000)

Year	All-Cause Mortality	Mental-health Mortality
1980	1386.6	8.5
1985	1443.8	12.5
1990	1330.6	14.2
1995	1210.4	19.9
2000	1039.7	17.9
2005	965.8	18.5
2010	854.9	20.2
2015	801.8	28.0
Change	− 42.1%	228.4%

Source: OECD Health Status: Causes of Mortality

As a result, mental health is making up an increasingly large proportion of the burden of disease. 28 of the 36 OECD countries have seen an increase in mental health deaths between 1995 and 2015, with the UK experiencing the highest increase from 18.2 to 67 deaths per 100,000 population. Numerous country-specific studies support these claims, showing that more recent cohorts have more mental health problems in the US [3], the UK [4], Belgium [5], Italy [6] and Sweden [7]. As a result, mental health problems are now the third greatest health burden in upper-middle and high-income countries [8] and a conservative calculation would make them the fifth highest global contributor to disability adjusted life years [9].

There is a wide variation in the number of deaths caused by mental and behavioural disorders among OECD countries. Before 1985, the furthest outlier every year is Israel, possibly because of conflict in the Middle East and trauma due to memory of the Holocaust [10]. After 1986, Finland is the only outlier, due to recording a far higher proportion of deaths from dementia than any other country. Despite the variation, there is a clear upward trend suggesting that mental health will continue to become a more pressing public health issue. Health policies and the allocation of government expenditure need to address the growing burden of mental illness.

The social determinants of health have a particularly great effect on the number and severity of mental health disorders, and social spending more directly addresses the social determinants of health than does spending on healthcare [11].

However, mean spending on social services across the OECD has risen from 16.5% of GDP to 20.5% since 1990, an increase of 24%, whilst spending on healthcare has increased from 6.5% to 8.8% of GDP, an increase of 35%. Different countries, however, have spent proportionately more than the average on healthcare spending, while others have prioritized social services. This creates an opportunity to determine whether different national priorities with respect to health and social spending are associated with differences in the mortality rates associated with mental health disorders.

Recent studies in the US [12], Canada [13] and across the OECD [14] suggest that increased social spending relative to health spending is associated with better outcomes across a variety of population health measures. However, no study has focussed on the relative contributions of health and social spending to death associated with mental health outcomes. We hypothesise that a higher ratio of expenditure on social services to direct healthcare spending will be associated with better mental health outcomes.

## Methods

### Study design

We conducted a cross-section and time-series study of panel data from the 36 OECD countries for a period of 22 years from 1995 and 2016. The data for this analysis was gathered from the publicly available time series data from OECD.stat [15]. This period was chosen as it was the most recent 22-year period for which complete and reliable country-level longitudinal data has been published by the OECD on the healthcare expenditure, social expenditure and the health outcomes of interest. Using these, it is possible to make comparisons across countries and across years. With some countries missing some data for particular years, 603 observations out of a possible 792 were recorded, which is a completeness rate of 76%. It appears that the missing data is random.

The Health Research Ethics Board at the University of Manitoba deemed this study exempt from ethics review as it is a secondary analysis of publicly available data.

### Dependent variables

The dependent variable is a systematically defined crude mortality rate per 100,000 due to “mental and behavioural disorders”, including substance abuse and alcoholism. Using this broad range will give a rounded view of a population mental health and provide more thorough understanding of the effects of changing government spending patterns [16].

The World Health Organization (WHO) maintains the International Classification of Diseases (ICD), which is the foundation for the identification of health trends and statistics globally. ICD systematically defines the universe of diseases, disorders and conditions. Since 1967, member states of the WHO have reported mortality and morbidity statistics using the latest revision of the ICD so that international comparisons are feasible. The system is periodically revised to reflect changes in health-related knowledge, and when the system is revised, WHO member states perform a "bridge-coding" exercise in which deaths are dual coded according to both the outgoing and incoming revisions. In this way, comparability ratios can be calculated for each cause. ICD-10, the 10^th^ revision of the system, was introduced in 1990 and chapter v deals specifically with “Mental and Behavioural Disorders”, coded as F00 to F99, which are defined as psychiatric conditions expressed primarily as abnormalities of thought, feeling, and behaviour that produce distress or impair function. High-level classifications of mental and behavioural disorders are identified in Table 2.

**Table 2:** ICD-10 Index for Mental and Behavioural Disorders (F00-F99)

Code	Mental or Behavioural Condition
F00 – F09	Mental disorders due to known physiological conditions (eg. Alzheimer’s, F00; Vascular dementia, F01)
F10 – F19	Mental and behavioural disorders due to psychoactive substance use
F20 – F29	Non-mood psychotic disorders (eg. Schizophrenia, F20)
F30 – F39	Mood (affective) disorders
F40 – F48	Anxiety and other non-psychotic mental disorders
F50 – F59	Behavioural syndromes associated with physiological disturbances and physical factors (eg. Eating disorder, F50; Sleep disorder, F51)
F60 – F69	Disorders of adult personality and behaviour
F70 – F79	Intellectual disabilities
F80 – F89	Pervasive and specific developmental disorders (eg. Specific developmental disorders of speech and language, F80)
F90 – F98	Behavioural and emotional disorders with onset usually during childhood and adolescence (eg. Attention deficit hyperactivity disorder, F90; Conduct disorder, F91)
F99	Unspecified mental disorder (disorder not otherwise specified)

Source: World Health Organization https://icd.who.int/browse10/2016/en (accessed 4 November 2019)

The dependent variable is defined as the crude mortality rate per 100,000 due to mental and behavioural disorders, as defined by ICD-10, F00 – F99.

### Independent variables

We used three variables to capture different aspects of social and healthcare spending – social expenditure as a proportion of GDP, healthcare expenditure as a proportion of GDP and the ratio or social to healthcare expenditure. Each was measured in US dollars (USD) at constant prices and constant Purchasing Power Parity (PPP) (reference year: 2010) to control for variations in the exchange rate and inflation between countries over the period.

Social expenditure as a proportion of GDP was extracted from the ‘Social Expenditure – Aggregated Data’ table of OECD.stat, which draws from the OECD Social Expenditure Database (SOCX) [17].

Social spending encompasses all universal and means-tested transfers from the government to the population. This includes old age benefits, survivors’ benefits, incapacity benefits, family benefits, unemployment benefits, in-work welfare payments and housing subsidies. Some of these are cash transfers and others are subsidies, such as subsidised social housing, or benefits-in-kind, such as incapacity rehabilitation services. It does not include expenditure such as education, law enforcement, public transportation etc which may come under a broader definition of ‘social spending’.

Healthcare expenditure as a proportion of GDP includes provision of all curative services, long-term care, ancillary services, medical goods, preventative care and administration. This was extracted from the ‘Heath Expenditure and Financing’ table in OECD.stat. This utilises the Joint Health Accounts Questionnaires, which bring together data from the OECD, WHO and the European Statistical Office (EUROSTAT).

The ratio of social spending to healthcare spending has been calculated using these.

Our models also control for a number of independent variables, based on both the review of the literature and the availability of data from OECD databases. Demographic differences were accounted for by controlling for the gender and age distributions in each country. Gender was accounted for using the female to male ratio and age uses the proportion of the population in each of five age groups (0-14, 15-29, 30-44, 45-64 and 65+). Wealth disparities between countries were controlled for by using GDP per capita (2010 constant prices and constant PPPs, US dollars), which was transformed into log GDP per capita to allow for more efficient estimates. The unemployment rate was included to control for a commonly perceived external contributor to mental health problems [18]. Finally, year and country variables were included to control the influence of aggregate (time series) trends over 21-year time period and variations across 36 OECD member countries on outcome measures of interest.

### Statistical analysis

We estimated 6 different models using the linear random effects model, each of which considers healthcare and social expenditure variables in slightly different ways. We anticipated two statistical issues to challenge our estimates – collinearity between social and health spending, and a complex relationship between healthcare spending and the dependent variable. The first problem could occur because there is a strong correlation between the levels of healthcare and social spending in any country, which might lead to inefficient estimates and statistically insignificant results. The second problem could occur because the level of healthcare spending both determines and responds to levels of morbidity and mortality in any country. On the one hand, increased healthcare expenditure might be associated with lower mortality rates, but higher mortality rates might call forth additional healthcare spending. Our six models attempt to clarify the effects of healthcare and social spending on mortality rates in the wake of these statistical issues.

While we used economic theory to choose the variables to be included in our models, the most efficient estimation technique is largely determined by the characteristics of the data itself. We have 22 years of data over 36 countries. We would expect data in any one country to follow patterns specific to that country, which may be more or less distinct from other countries. Similarly, global effects such as recessions will affect all countries in any one year, but will not necessarily have identical effects in each country. Therefore, we want to use an estimation technique that allows for these kinds of differences, but at the same time to use a model that generates the most efficient estimates for our data. Two statistical tests allow us to confirm that the random effects model optimizes the trade-off between efficiency and flexibility – the Hausman test and Akaike Information Criterion estimation (Akaike test).

All statistical analyses were conducted using STATA statistical software version 15.0. For each regression model, a goodness of fit test was conducted to determine how well the model fit the selected data.

## Results

We estimated six models to achieve a fuller understanding of the relationship between healthcare spending, social spending, the ratio of these two types of expenditure, and mental health outcomes (Table 3). All models controlled for age, gender, log GDP per capita and the unemployment rate, and included country effects and year effects. In each case, we used *p*<.05 to determine significance.

**Table 3:** Modelling the effects of spending patterns on mental health outcomes

Coeff (SE) *P*-Value *p < .05	MODEL 1	MODEL 2	MODEL 3	MODEL 4	MODEL 5	MODEL 6
Health exp	−48.0 (40.8) 0.239	N/A	− 109.4 (48.8) 0.025*	128.5 (50.6) 0.011*	N/A	445.6 (119.0) 0.000*
Social exp	N/A	− 82.1 (17.9) 0.000*	N/A	− 117.8 (23.2) 0.000*	− 77.0 (18.8) 0.000*	− 236.0 (46.3) 0.000*
Social exp./ health exp. ratio	N/A	N/A	−4.6 (1.6) 0.004*	N/A	−1.4 (1.3) 0.287	9.1 (3.1) 0.003*
Female to male ratio	15.3 (173.0) 0.929	85.3 (167.6) 0.611	22.5 (174.8) 0.898	58.0 (172.3) 0.736	45.6 (173.1) 0.792	89.4 (171.5) 0.602
Age 15–29	− 166.6 (43.0) 0.000*	− 159.2 (37.4) 0.000*	− 153.9 (43.2) 0.000*	−174.4 (42.5) 0.000*	− 166.1 (42.7) 0.000*	− 204.7 (43.4) 0.000*
Age 30–44	− 387.0 (47.9) 0.000*	− 355.0 (40.6) 0.000*	− 347.4 (48.9) 0.000*	− 348.0 (47.2) 0.000*	− 352.3 (48.2) 0.000*	− 382.0 (48.3) 0.000*
Age 45–64	− 238.5 (39.4) 0.000*	− 244.7 (35.5) 0.000*	− 222.0 (40.3) 0.000*	− 245.0 (38.3) 0.000*	− 238.8 (39.5) 0.000*	− 298.9 (42.2) 0.000*
Age 65 +	− 265.9 (45.5) 0.000*	− 229.8 (43.2) 0.000*	− 220.9 (47.2) 0.000*	− 223.9 (44.9) 0.000*	− 221.8 (45.9) 0.000*	− 268.7 (47.1) 0.000*
Log GDP	−5.6 (5.1) 0.271	−6.0 (4.7) 0.196	−4.8 (5.0) 0.341	− 6.8 (5.0) 0.169	−6.2 (5.0) 0.213	− 6.1 (4.9) 0.212
Unemploy-ment Rate	−0.1 (0.1) 0.480	0.2 (0.1) 0.113	0.2 (0.1) 0.262	0.3 (0.1) 0.027*	0.3 (0.1) 0.075	0.3 (0.1) 0.040*
Year	1.5 (0.1) 0.000*	1.6 (0.1) 0.000*	1.5 (0.1) 0.000*	1.6 (0.1) 0.000*	1.6 (0.1) 0.000*	1.7 (0.1) 0.000*
Constant	− 2796.0 (316.5) 0.000*	− 3039.0 (288.6) 0.000*	− 2658.6 (310.3) 0.000*	− 2840.4 (306.2) 0.000*	− 2885.86 (314.0) 0.000*	− 3074.0 (314.3) 0.000*

Source: author’s calculations. Standard errors are reported in parentheses. * indicates significance at the 95% level

Model 1 omits social spending and includes only healthcare spending. Our results demonstrate no statistically significant relationship between the level of healthcare spending and death rates for mental and behavioural disorders when the level of social expenditure is not controlled for.

Model 2 considers only social spending and omits healthcare spending. Results indicate that higher social spending is strongly associated with a reduction in the death rate, even without controlling for the level of healthcare spending.

Model 3 is the complete model, in which both healthcare spending and the ratio of social to healthcare spending are included. Results demonstrate a significant association between higher levels of health spending and lower crude death rates from mental and behavioural disorders, but also that there is a significant association between a higher ratio of social spending to healthcare spending and lower crude death rates from mental and behavioural disorders. Note that healthcare spending is measured as a proportion of GDP and therefore a one unit increase in healthcare spending would be equivalent to increasing healthcare spending by 1% of GDP – from, for example, 8% to 9% of GDP. By contrast, a one-unit increase in the ratio of social spending to healthcare spending represents a very much smaller monetary amount. Therefore, directly comparing the size of the coefficients is misleading.

Model 4 includes both healthcare spending as a proportion of GDP and social spending as a proportion of GDP independently, and excludes the ratio between the two. In this case, both healthcare and social spending are statistically significant (*p*<.05), yet increased healthcare spending is associated with higher death rates due to mental and behavioural issues, while higher social spending is associated with lower death rates.

Model 5 includes social expenditure as a proportion of GDP and the ratio of social to health expenditure. The latter variable is not significant, while the first is significant and shows that higher social spending is associated with lower death rates.

Finally, model 6 includes all three financial variables – social expenditure as a proportion of GDP, health expenditure as a proportion of GDP and the ratio of social to health expenditure. All three variables are statistically significant (*p*<.05) although only higher social expenditure is associated with a reduction in the crude death rate. Both increased healthcare spending and a higher ratio of social to health expenditure are associated with significant increases in the crude death rate, contrary to expectation.

We expected both healthcare and social expenditure to be negatively associated with mental health mortality rates, but we anticipated that statistical challenges would make it difficult to estimate their relative effects in a straightforward way.

When healthcare spending alone is considered, it is not significantly associated with death rates due to mental and behavioural disorders. This is, in fact, the outcome we would expect if healthcare expenditure automatically responds, at least in part, to increased morbidity and mortality. The direction of the association is ambiguous. By contrast, there is a strong negative association between the level of social spending and mortality outcomes when healthcare spending is omitted, but a complete model should allow both healthcare and social spending to play a role.

We also expected there to be a strong correlation between healthcare expenditure and social expenditure, because countries that spend a lot on one also tend to be relatively generous spenders on the other. This collinearity makes it difficult to distinguish, statistically, between the effects of the two variables, leading to imprecise estimates and counter-intuitive signs as in models 4 and 6, both of which suggest that spending more on healthcare will be associated with increases in the mental health mortality rate.

As a consequence of these two statistical challenges, we substituted the ratio of social to health expenditure for the level of social expenditure in model 3, and for the level of healthcare expenditure in model 5. Both of these estimates confirm the importance of social spending, although model 5 allows no statistically significant role for healthcare spending or for the ratio of social to healthcare spending, in effect reverting to the incomplete model 2. For all these reasons, model 3 is the best estimate of the impact of healthcare and social expenditure on mental health mortality rates.

Finally, the Akaike and Hausmann tests confirmed our choice of random effects model.

## Discussion

Previous research has demonstrated the importance of the social determinants of health and how social spending may be the most effective way for developed countries to improve health outcomes in measures as diverse as adult obesity, asthma, lung cancer, type 2 diabetes, infant mortality and life expectancy.^11 13^ This article addresses the important area of mental health by measuring outcomes by crude mortality rates for "mental and behavioural disorders".The results of our models confirm our hypothesis.

We find that healthcare spending alone is not significantly associated with death rates due to mental and behavioural disorders, but that higher levels of social spending are significantly associated with better mental health in the population, as is a higher ratio of social spending to healthcare spending. We expected social determinants to be particularly relevant for mental health outcomes and our model supports this, demonstrating that higher social spending, when controlling for the level of healthcare spending per capita, is significantly associated with lower mortality rates. When allocating additional funding, a decision to allocate funding to social as opposed to healthcare spending would be expected to lead to improved population mental health.

Using mortality rates makes this conclusion and further expectations more robust as the analysis is able to largely mitigate the measurement problem of cultural differences between countries that might influence the way that less concrete measures of mental health are reported [19]. The level of stigma surrounding mental health problems varies between cultures which may lead to inconsistent levels of reporting as different proportions of individuals will refuse to disclose their mental health problems. This stigma is strong enough to prevent people seeking medical help as this admission of poor mental health evokes feelings of shame [20]. Using mortality figures is more likely to avoid these problems than other measurable variables because the cause of death tends to be a clearer variable to measure. It is true that the reporting of deaths due to mental health issues is by no means perfect – studies show consistent underreporting in deaths due to mental health too [21, 22]. Difficulties with classification and coding can add to this underreporting. However, despite these concerns, it is unlikely that errors in official reports of the cause of death would affect the statistics as much as widespread under-reporting from individuals trying to adhere to cultural norms. Therefore, the use of mortality rates and OECD classifications gives the best possible representation of the distribution of mental health deaths available to us.

This analysis shows how the differences in the proportions of GDP per capita that countries allocate to social services and healthcare are significant in determining the mental health of their populations, adding to the growing body of literature examining the relationship between health outcomes and social expenditure. Hence, for OECD countries, increasing the ratio of social spending to healthcare spending may lead to better mental health outcomes than increasing healthcare spending alone. This means that population mental health could be improved as a deliberate side effect of a policy with a social aim, making such policies more politically appealing - e.g. improving housing subsidies can simultaneously reduce homelessness and improve mental health. Given that the social determinants of mental health are so broad, it would seem that there is a lot of scope for welfare policies to reap this additional benefit.

The importance of mental health in developed nations has been recently brought to popular attention with the analysis of Anne Case and Angus Deaton concerning “deaths of despair” [23]. Their analysis suggests that the mortality rate for white, middle-aged Americans without college education is increasing, a trend that the authors suggest may be soon to emerge in other developed nations, including Britain, Ireland and Canada [24]. This trend is the result of the increase in “deaths of despair” (deaths due to suicide, drug poisoning and alcohol-related liver failure) being greater than the reduction in mortality rates due to advances in medical science since the phenomena began in 1998. This despair, the authors claim, is brought about by the “cumulative disadvantage” suffered by the afflicted demographic who are materially worse off than their parents, suffer from declining wages and few job opportunities, have historically low levels of social capital, experience a high rate of relationship breakdowns and high parental instability [23]. The most effective way of improving the mental health of this vulnerable group may be to remove the reasons for their despair. This means engaging in additional social spending, rather than relying on additional healthcare spending alone to address the observed increase in mortality rates.

### Limitations

There are some limitations to this analysis that should be noted. Firstly, our choice of outcome variable means that only mental health issues that are severe enough to result in fatalities are accounted for. The vast majority of mental health problems are not this severe, even though they may have a significant impact on an individual’s quality of life. The WHO estimates that 450 million people worldwide live with a mental illness [25], but mortality has been calculated at around 8 million per year [26]. Analysis of other measures of mental health, such as the prevalence of particular conditions, would be a possible future avenue to explore in this topic. Additionally, this paper uses the statistics for death from mental health according to the OECD, however, different measures dispute the classification of mental health deaths. The Emerald Policy Report argues that the distinctions between problems due to mental illness and problems resulting from neurological disorders, self-injury, musculoskeletal disorders are not clear and that the contribution of mental illness to other causes of death is often underappreciated. Replicating this paper using other definitions of mental illness would lend further support.

Secondly, this analysis only uses OECD countries, meaning that these results should only be taken to apply to high-income countries. There may be a certain level of healthcare spending per capita that must be reached before the ratio of social to healthcare spending begins to favour social spending, but this is not something we have investigated here. Thirdly, this paper groups all welfare transfer payments under ‘social spending’ and this is a somewhat imprecise measure. Future analysis could investigate whether the effect noted is only due to certain transfers within that group, or whether other forms of social spending (e.g. education) could have an even greater effect.

## Conclusion

The past 100 years have seen enormous strides made in improving the physical health of populations through innovations and investment in medical technology and healthcare facilities. However, the same cannot be said for mental health, which has increased its share of the global burden of disease. This is an indication that the approach that has been so successful for physical health does not translate into equally successful outcomes for mental health and that a different approach is necessary. Our results confirm this, finding that higher levels of social spending are more closely associated with lower levels of deaths from mental health problems than higher levels of healthcare spending. Indeed, other authors have shown that some physical health outcomes are now in the same position, suggesting that developed countries have reached the point where the diminishing returns to healthcare spending mean that policies aiming to improve population health are better off focussing their efforts on improving their social spending programs than funnelling more money directly into healthcare. We hope to have strengthened this argument with our findings.

## Data Availability

The datasets generated during and/or analysed during the current study are available in the OECD Statistics repository, https://stats.oecd.org/ (OECD. OECD Statistics [Internet]. Paris: OECD.Stat (database); 2019 [cited 30 June 2019]. Available from https://stats.oecd.org/#.

## Abbreviations

GDP: Gross Domestic Product is a monetary measure of the market value of all the final goods and services produced by a country in a given year
Eurostat: (European Statistical Office) is located in Luxembourg. Its main responsibilities are to provide statistical information to the institutions of the European Union (EU) and to promote the harmonisation of statistical methods across its member states and candidates for accession as well as European Free Trade Association countries
ICD-10: ICD-10 is the 10th revision of the International Statistical Classification of Diseases and Related Health Problems (ICD), a medical classification list maintained by the World Health Organization (WHO)
OECD: The Organisation for Economic Co-operation and Development is an intergovernmental economic organisation with 36 member countries, founded in 1961 to stimulate economic progress and world trade
PPP: Purchasing Power Parity measures prices in different countries using a common basket of goods and services
SOCX: SOCX is the OECD Social Expenditure Database
USD: United States dollars
WHO: The World Health Organization (WHO) is a specialized agency of the United Nations, established in 1948, that monitors international public health
